# Development and Pilot Evaluation of a Training-of-Trainers Model for School-Based Sexuality Education Within the ESPRIT Project

**DOI:** 10.3390/ijerph23070843

**Published:** 2026-06-26

**Authors:** Alessandra Casuccio, Nicolò Piazza, Giada Cordova, Patrizia Ferro, Nazareno Inzerillo, Alessio Castiglione, Manola Comar, Barbara Suligoi, Maria Cristina Salfa, Daniele Gianfrilli, Franz Sesti, Silvia Gazzetta, Laura Brunelli, Palmira Immordino, Vincenzo Restivo, ESPRIT Study Collaboration Group

**Affiliations:** 1 Department of Health Promotion, Mother and Child Care, Internal Medicine and Medical Specialities, University of Palermo, 90127 Palermo, Italy; nicolapiazza.07@gmail.com (N.P.); cordovagiada@gmail.com (G.C.); pattyferro91@gmail.com (P.F.); nazareno.inzerillo@outlook.it (N.I.); alessio.castiglione@unipa.it (A.C.); palmira.immordino@unipa.it (P.I.); 2 Department of Advanced Translational Microbiology, Institute for Maternal and Child Health, Istituto di Ricovero e Cura a Carattere Scientifico (IRCCS) “Burlo Garofolo”, 34137 Trieste, Italy; manola.comar@burlo.trieste.it; 3 Department of Medical, Surgical, and Health Sciences, University of Trieste, 34127 Trieste, Italy; 4 National AIDS Unit, Department of Infectious Diseases, Italian National Institute of Health, 00161 Rome, Italy; bsuligoi@gmail.com (B.S.); mariacristina.salfa@iss.it (M.C.S.); 5 Department of Experimental Medicine, Sapienza University of Rome, 00185 Rome, Italy; daniele.gianfrilli@uniroma1.it (D.G.); franz.sesti@uniroma1.it (F.S.); 6 Complex Operational Unit Accreditation, Quality, and Clinical Risk, Friuli Centrale Healthcare University, 33100 Udine, Italy; silvia.gazzetta@asufc.sanita.fvg.it (S.G.); laura.brunelli@asufc.sanita.fvg.it (L.B.); 7 Department of Medicine, University of Udine, 33100 Udine, Italy; 8 Department of Medicine, Kore University of Enna, 94100 Enna, Italy; vincenzo.restivo@unikore.it

**Keywords:** sexuality education, public health training, health education, training of trainers, capacity building

## Abstract

**Highlights:**

**Public health relevance—How does this work relate to a public health issue?**

- Sexuality education is inconsistently implemented in Italian schools despite its importance for adolescent sexual health promotion.
- This study describes a structured multidisciplinary Training-of-Trainers model developed to support school-based sexuality education pathways.

**Public health significance—Why is this work of significance to public health?**

- The programme addresses the need for scalable and evidence-informed approaches to strengthen educator capacity in sexuality education.
- Trained participants showed higher short-term knowledge scores compared with untrained advanced public health residents.

**Public health implications—What are the key implications or messages for practitioners, policy makers and/or researchers in public health?**

- Structured Training-of-Trainers programmes may support implementation of coordinated sexuality education interventions in school settings.
- Further research is needed to evaluate long-term effectiveness, sustainability, and transferability of this model in real-world public health practice.

**Abstract:**

Background: Sexuality education is essential for adolescent health and well-being, yet in Italy it is not included in a mandatory national curriculum, resulting in heterogeneous implementation across regions. Within the ESPRIT project, a multidisciplinary training-of-trainers (ToT) model was developed to prepare professionals to support school-based peer-education pathways. This study aimed to describe the training model and perform a pilot evaluation of short-term knowledge outcomes among trained participants. Methods: A pilot non-randomized controlled comparative study was conducted within the ESPRIT project framework. A multidisciplinary Training Team developed a structured ToT pathway based on WHO guidance, national recommendations, and peer-education models. Ten advanced public health residents in Hygiene and Preventive Medicine attended a three-day residential training course. One month later, a 10-item knowledge questionnaire was administered to trained participants (*n* = 10) and untrained advanced public health residents (*n* = 10). Results: Trained participants achieved higher questionnaire scores than the comparator group (median score 8 [IQR 2] vs. 3.5 [IQR 2]; *p* < 0.0005). Conclusions: Structured ToT programmes may represent a promising approach for strengthening professional preparation in sexuality education. Larger studies with longer follow-up are needed to evaluate sustainability and real-world implementation.

## 1. Introduction

Sexuality education represents a fundamental component of adolescent health and well-being. During adolescence, individuals experience important biological, psychological, and social changes that influence identity development, relationships, and sexual behavior. In this context, comprehensive sexuality education (CSE) has been widely recognized as an important strategy to support young people in developing the knowledge, skills, and attitudes necessary to make informed decisions about their health and relationships. CSE addresses multiple domains, including gender identity, emotional and relational competence, reproductive health, contraception, and prevention of sexually transmitted infections (STIs). Schools are widely recognized as key settings for health promotion and prevention programmes [1].

In Italy, sexuality education is not included in a mandatory national curriculum and educational activities are often implemented through locally developed initiatives involving schools, healthcare professionals, external experts, and public health institutions. As a result, substantial variability exists in both content and delivery across regions and schools [2]. Regional disparities persist, with fewer structured programs available in southern regions, representing an important public health challenge [2].

International organizations have consistently emphasized the importance of CSE as a core component of adolescent health promotion and well-being. Structured and equitable access to sexuality education has been associated with improved health literacy, informed decision-making, and the promotion of healthy relationships, supporting the need for consistent educational opportunities across different settings and populations [1,3].

According to the World Health Organization, sexual health is defined as a state of physical, emotional, mental, and social well-being in relation to sexuality [4]. STIs remain a major global public health issue, with millions of new infections occurring each year [5,6]. Many STIs are asymptomatic or underdiagnosed, and some infections increase susceptibility to Human Immunodeficiency Virus (HIV) acquisition and transmission [7,8,9]. Adolescents are particularly vulnerable due to biological susceptibility, behavioral risk factors, and limited access to reliable sexual health information [10,11,12,13]. In recent years, particularly following the COVID-19 pandemic, adolescents have increasingly relied on online sources and social media for information related to sexuality and sexual health [9,10]. Studies suggest that embarrassment, stigma, and lack of confidential access to professional counseling contribute to this trend, highlighting the need for structured and accessible sexuality education programmes within school settings [10,11,12,13,14].

In settings where sexuality education is not systematically embedded within school curricula, the preparation of adequately trained professionals becomes particularly important. Training-of-Trainers (ToT) approaches have been successfully applied in different public health and healthcare contexts to support capacity building, dissemination of knowledge, and standardization of educational interventions. Similarly, peer education models have demonstrated potential in promoting adolescent health literacy and engagement when supported by structured training pathways and appropriately prepared facilitators [15,16,17,18,19]. Peer education approaches are based on the premise that young people may be particularly receptive to information and behavioural messages delivered by trained peers who share similar social and developmental experiences. Previous studies have shown that peer-led interventions can contribute to improving adolescent health literacy, engagement, and sexual health knowledge when supported by structured training programmes and appropriate supervision of peer educators [15,16,17].

To address gaps in sexuality education and strengthen prevention of STIs, the Italian Ministry of Health funded the ESPRIT project (“Education in lower and upper secondary schools and support of the network of adolescent reference persons for the prevention of HPV and other sexually transmitted infections”) [20], which aims to assess knowledge, attitudes, and behaviors related to STIs among students, teachers, and parents, and to implement targeted educational interventions across different Italian regions.

Although STI and HPV prevention represent central objectives of the ESPRIT project, the training programme adopted a broader comprehensive sexuality education perspective, consistent with WHO and UNESCO recommendations. Accordingly, the programme addressed not only sexual and reproductive health topics but also communication skills, inclusivity, gender-related concepts, and sociocultural dimensions relevant to adolescent sexuality education [3].

Within this framework, training professionals capable of supporting peer education pathways represents a key step. The training-of-trainers (ToT) model is increasingly used in public health and educational contexts to promote scalable and sustainable knowledge transfer. However, evidence regarding structured ToT approaches specifically applied to sexuality education training remains limited, particularly in the Italian context.

To support the implementation of peer education pathways within the ESPRIT project, a multidisciplinary ToT training programme was developed. Given the exploratory nature of this initiative, a pilot evaluation was conducted to assess short-term knowledge outcomes among trained participants.

Advanced public health residents were selected as a comparator group because they represent healthcare professionals with academic exposure to preventive medicine and public health topics but without specific training in sexuality education or peer education methodologies. This allowed comparison with a group expected to have general health knowledge but not the specific competencies targeted by the training.

Accordingly, this study was conducted in two sequential phases. Phase 1 consisted of the development of a multidisciplinary Training-of-Trainers (ToT) programme aimed at preparing advanced public health residents to support sexuality education activities in secondary schools within the ESPRIT project. Phase 2 consisted of a pilot non-randomized comparative evaluation designed to explore short-term knowledge outcomes among trained participants compared with a group of untrained advanced public health residents.

The objectives of this study were: (i) to describe the development and implementation of a multidisciplinary Training-of-Trainers programme for school-based sexuality education within the ESPRIT project and (ii) to conduct a pilot non-randomized comparative evaluation of short-term knowledge outcomes among trained and untrained participants.

The study addressed the following research questions:

How can a structured multidisciplinary Training-of-Trainers programme be developed to support sexuality education activities in secondary schools within the ESPRIT project framework?

Do participants who complete the ToT programme achieve higher short-term sexuality education knowledge scores than comparable participants who did not attend the training?

For the evaluation phase, we hypothesized that participants who completed the ToT programme would achieve higher knowledge questionnaire scores than untrained participants.

## 2. Materials and Methods

### 2.1. Study Design and Ethical Approval

This study comprised two sequential phases. Phase 1 involved the development and implementation of a multidisciplinary Training-of-Trainers (ToT) programme within the ESPRIT project. Phase 2 consisted of a pilot non-randomized comparative evaluation of short-term knowledge outcomes among trained and untrained participants.

The project was approved by the Ethics Committee of Friuli Venezia Giulia (CEUR-2023-Sper-34; approval date: 26 September 2023). In addition, ethical authorization was obtained from the Local Ethics Committee of Palermo 1 (protocol number 05/2023; approval date 14 November 2023), in accordance with national regulations governing multicenter research projects.

The study was conducted in accordance with the Declaration of Helsinki and local institutional requirements. Written informed consent was obtained from all participants before study participation.

### 2.2. Setting, Study Period and Study Duration

The study was conducted within the ESPRIT project network across three Italian regions (Friuli Venezia Giulia, Lazio, and Sicily). The residential training course was delivered in Palermo (Sicily).

The training course took place in February 2024, and questionnaire administration was conducted one month after completion of the training.

### 2.3. Development of the Training Programme

The training programme was directly delivered by members of the Training Team (TT1), including professionals with complementary backgrounds in public health and preventive medicine, child and adolescent neuropsychiatry, pedagogy, and communication. All facilitators had previous experience in sexuality education activities conducted in school settings and local training centres, as well as experience in health promotion and educational interventions targeting adolescents. The multidisciplinary composition of the team was considered essential to address the biological, psychological, educational, and social dimensions of sexuality education. The TT1 team included two public health residents, a general practitioner, a pedagogist, a communication expert, and a child and adolescent neuropsychiatry resident.

The development process was informed by multiple complementary sources, including the ESPRIT project protocol [20], WHO International Technical Guidance on Sexuality Education [3], and the STASH peer-education model [15].

The TT1 conducted a structured review and discussion of these resources to identify key educational domains, competencies, and training approaches relevant to sexuality education in secondary schools. Through iterative meetings and consensus-based discussions, the team selected training content and learning activities considered most appropriate for the objectives of the ESPRIT project and the Italian school context.

The resulting programme integrated scientific knowledge on sexual and reproductive health, communication skills, peer education methodologies, inclusivity and gender-related concepts, group facilitation techniques, and project planning competencies. Learning objectives were defined to support both knowledge acquisition and the development of practical skills required for future school-based educational activities.

Through comparative evaluation of these frameworks, a training model was developed consisting of three main phases:

- Data collection and problem analysis;
- Training-of-trainers programme, including group-building and development of a final project;
- Presentation and adaptation of the final project for implementation in participating schools.

The training followed WHO international sexuality education domains, including relationships, values and rights, gender understanding, violence prevention, health and well-being skills, human body and development, sexuality and behavior, and sexual and reproductive health.

Senior scientific coordinators with expertise in public health and project supervision participated as external observers during the training course.

The programme was delivered as a three-day residential course corresponding to a total of 24 training hours (8 h per day). The training combined theoretical sessions, experiential learning activities, peer education methodologies, group facilitation techniques, sexuality education content, and project planning exercises. A detailed description of the training activities, educational methods, and learning objectives for each training day is provided in Supplementary Tables S1–S3.

The three-day residential format was selected to promote intensive learning, multidisciplinary interaction, team-building activities, and active participation. The duration was determined by the project team as a pragmatic balance between feasibility, participant availability, and the need to cover the core educational domains identified during programme development.

No formal training needs assessment was conducted prior to programme development. However, the content selection process was informed by the objectives of the ESPRIT project, existing evidence on adolescent sexual health education, and the multidisciplinary expertise of the development team.

A formal consensus methodology was not employed. Training contents were selected through multidisciplinary discussions and agreement among members of the Training Team.

### 2.4. Participants and Selection Procedures

Participation in the training programme was based on voluntary enrollment following invitation within the ESPRIT project professional network. No formal selection based on motivation scoring or prior competencies was performed.

### 2.5. Experimental Group

The experimental group consisted of ten advanced public health residents who attended the three-day residential ToT training and were selected as future trainers within the ESPRIT project. Participants for the Training-of-Trainers programme were recruited by the Sicilian operational unit of the ESPRIT project, coordinated by the Section of Hygiene and Preventive Medicine of the University of Palermo. A purposive sampling strategy was adopted to identify future trainers with backgrounds relevant to public health and health promotion activities.

Eligible participants included advanced public health residents (third and fourth year) in Hygiene and Preventive Medicine interested in sexuality education and school-based health promotion. Participation was voluntary. Individuals unable to attend the entire residential programme were not considered eligible.

The final training group included 10 participants (5 males and 5 females) with a mean age of 28 years. The selection process aimed to create a multidisciplinary group of future trainers sharing a common background in public health and preventive medicine.

### 2.6. Comparator Group

For the pilot evaluation phase, the comparator group consisted of third- and fourth-year residents in Hygiene and Preventive Medicine from the same academic setting who had not participated in the Training-of-Trainers programme. This group was selected because participants shared a comparable educational background in public health and preventive medicine but had not received the specific sexuality education training delivered within the ESPRIT project.

### 2.7. Eligibility Criteria

Inclusion criteria for the experimental group included:

- Participation in the residential training course;
- Professional involvement within the ESPRIT project network.

Inclusion criteria for the comparator group included:

- Enrollment in a public health residency programme;
- No participation in the ToT training programme.

### 2.8. Training Intervention

Participants attended a three-day residential training course designed to:

- Strengthen communication and facilitation skills with adolescents;
- Introduce laboratory-based and interactive educational activities;
- Provide foundational knowledge in sexuality education;
- Expand conceptual understanding of sexuality beyond biomedical aspects.

### 2.9. Questionnaire Development and Outcome Assessment

The questionnaire was developed specifically for the pilot evaluation and was intended to assess participants’ familiarity with selected domains addressed during the Training-of-Trainers programme rather than to provide a comprehensive measure of sexuality education knowledge.

Items were selected by the multidisciplinary Training Team to reflect key topics covered during the course, including sexual and reproductive health, STI prevention, HIV-related concepts, gender identity and diversity, inclusive sexuality education terminology, and contemporary adolescent sociocultural contexts. The questionnaire was therefore conceived as an exploratory educational assessment tool aligned with the content of the training programme.

The item concerning generational classification was not intended to assess gender concepts but rather participants’ familiarity with contemporary adolescent cohorts and sociocultural contexts, which were discussed during the training programme as part of communication strategies targeting young people. Formal psychometric validation was not performed, and results should be interpreted accordingly.

The primary outcome of the study was defined as the difference in questionnaire scores between trained and untrained participants at one-month follow-up.

To assess knowledge related to sexuality education and sexual health, a questionnaire consisting of 10 multiple-choice questions was developed (Table 1). The questionnaire was based on literature review, WHO training materials, and ESPRIT project educational objectives.

Due to the pilot nature of the study, formal psychometric validation was not performed. However, the questionnaire content was reviewed by TT1 members to ensure content relevance and consistency with training objectives.

Correct answers were scored as 1 and incorrect answers as 0.

### 2.10. Follow-Up Timing

The questionnaire was administered one month after completion of the residential course in order to evaluate short-term knowledge retention following training.

### 2.11. Data Collection and Anonymity

Questionnaires were administered in anonymous format. No identifying personal data were collected for analysis purposes.

### 2.12. Statistical Analysis

Questionnaire scores were entered into Microsoft Excel and expressed as median/interquartile range (IQR). Statistical comparisons between groups were performed using the Mann–Whitney U test. Differences in the proportion of correct answers for individual questionnaire items were evaluated using Fisher’s exact test.

Statistical analyses were conducted using IBM SPSS Statistics software version 24 (IBM Corp., Armonk, NY, USA). SPSS version 24 was the licensed statistical software available at the participating institution during the study period. A two-sided *p*-value ≤ 0.05 was considered statistically significant.

## 3. Results

### 3.1. Training Implementation

A total of 10 advanced public health residents participated in the three-day residential training course and completed all planned training activities.

The training programme was delivered as scheduled and covered group-building activities, peer education theoretical frameworks, and practical project development sessions aimed at preparing participants for school-based sexuality education interventions.

All participants completed the training and participated in the follow-up evaluation.

### 3.2. Participant Groups

The final analysis included 20 participants divided into two groups:

- Experimental group: 10 advanced public health residents who attended the ToT training;
- Comparator group: 10 advanced public health residents who did not attend the training.

### 3.3. Questionnaire Outcomes

One month after completion of the training programme, the 10-item knowledge questionnaire was administered to both groups (Table 1).

Trained participants achieved higher questionnaire scores than the comparator group (median score 8 [IQR 2] vs. 3.5 [IQR 2]; *p* < 0.0005), indicating higher knowledge scores among trained participants at follow-up.

### 3.4. Question-Level Performance

The frequencies and percentages of correct answers for each questionnaire item in both groups are reported in Table 1.

Overall, trained participants showed consistently higher correct response rates across most questionnaire items compared to the comparator group. The largest differences were observed in items assessing terminology, gender-related concepts, and contemporary sexuality education knowledge domains, suggesting greater familiarity with the topics addressed during the training programme.

## 4. Discussion

This pilot study describes the development and preliminary evaluation of a multidisciplinary training-of-trainers (ToT) programme implemented within the ESPRIT project, aimed at strengthening sexuality education delivery in secondary schools. The questionnaire was designed to reflect the multidimensional nature of the training programme, which extended beyond STI prevention alone and included broader sexuality education domains recommended by international sexuality education frameworks. Consequently, the instrument should be interpreted as an exploratory measure of familiarity with selected training topics rather than a validated measure of sexuality education competence.

The multidisciplinary content of the programme is consistent with international comprehensive sexuality education frameworks, which emphasize that sexuality education extends beyond STI prevention alone and includes relational, social, emotional, and diversity-related dimensions of sexual health [3].

The findings indicate that participants who attended the Training-of-Trainers programme achieved higher knowledge scores than the comparator group at follow-up. However, given the pilot design, absence of baseline assessment, potential selection bias, and use of a non-validated questionnaire, these differences should be interpreted cautiously and cannot be considered definitive evidence of programme effectiveness.

In the Italian context, where sexuality education is not currently included within a mandatory national curriculum and implementation remains heterogeneous across regions and schools, capacity-building initiatives targeting professionals involved in educational activities may represent a pragmatic strategy to improve the quality and consistency of sexuality education interventions.

The findings suggest that a structured ToT approach may represent a potentially useful strategy to support the preparation of professionals involved in school-based sexuality education and peer education pathways.

These findings are consistent with previous literature highlighting the potential value of structured training models and peer-education approaches in promoting adolescent sexual health and improving health education delivery [15,16,17,21,22,23,24,25,26].

Beyond knowledge acquisition, the training programme was designed to promote soft skills relevant to adolescent health education, including communication strategies, group facilitation, and collaborative problem-solving. Although these competencies were not formally measured, they represent key components of sexuality education programmes and are consistent with international recommendations emphasizing multidimensional training approaches.

From a broader implementation perspective, the study contributes a structured multidisciplinary Training-of-Trainers model that may support the preparation of professionals involved in school-based sexuality education initiatives. Such approaches may be particularly relevant in settings where sexuality education is not systematically embedded within formal educational curricula and where implementation depends on local initiatives and trained personnel. The multidisciplinary nature of the programme represents a potential strength of the model, as sexuality education intersects multiple domains, including public health, psychology, pedagogy, and social sciences, and requires sensitivity to cultural and developmental factors. The involvement of professionals with diverse expertise may support the development of more comprehensive and context-sensitive educational interventions. Team cohesion and shared training experiences may also contribute to improved implementation of sexuality education programmes in school settings. Previous studies have highlighted the importance of trust-building and consistent messaging in adolescent health promotion and preventive interventions [16,17,18,19,25,27,28,29].

Despite these promising preliminary findings, the results should be interpreted with caution. Several limitations should be acknowledged. First, the sample size was small and limited to a specific project context, reducing statistical power and limiting generalizability. Second, participation in the training programme was voluntary and the comparator group was selected based on professional profile rather than randomization, introducing the possibility of selection bias. Although both groups were recruited from a similar public health educational environment and included advanced residents in Hygiene and Preventive Medicine, participants selected for the Training-of-Trainers programme may have had greater motivation, interest in sexuality education, or previous exposure to related topics. Third, no pre-intervention assessment was performed. Consequently, baseline knowledge levels were not available, and it cannot be determined whether differences observed between groups were entirely attributable to participation in the training programme or were already present before the intervention. Furthermore, the questionnaire used to assess knowledge was specifically developed for project-related educational objectives and was not formally psychometrically validated. In addition, the instrument assessed multiple heterogeneous domains addressed during the training programme and was not designed to represent a single psychometric construct. Therefore, the findings should be considered exploratory and hypothesis-generating rather than confirmatory evidence of programme effectiveness.

Future studies should include larger samples, baseline assessments, validated measurement tools, and longer follow-up periods, including evaluation of training transfer into real school-based educational practice.

Despite these limitations, this study provides a preliminary description of a structured training model that may be adaptable to different geographical and institutional contexts. The use of a Training-of-Trainers approach is also consistent with previous experiences in public health and healthcare education, where such models have been employed to strengthen capacity building, improve dissemination of knowledge, and support the implementation of complex educational interventions [18,19].

The ToT approach may support the development of coordinated and multidisciplinary sexuality education initiatives, particularly in settings where standardized school-based programmes are not yet fully implemented.

Although knowledge represents an important prerequisite for sexuality educators, it constitutes only one component of effective educational practice. Competencies such as communication skills, relationship-building, facilitation abilities, and the capacity to create inclusive learning environments are also fundamental elements of comprehensive sexuality education delivery. While these dimensions were addressed during the training programme, they were not formally evaluated in the present pilot study.

Future research should therefore extend evaluation beyond knowledge outcomes and investigate the impact of Training-of-Trainers programmes on relational competencies, communication skills, educational practices, and real-world implementation of sexuality education activities in school settings.

Further research should focus on evaluating the long-term sustainability of this training model, its impact on educational practice in schools, and its potential influence on adolescent sexual health knowledge, attitudes, and behaviors.

## 5. Conclusions

This pilot study describes the development and preliminary evaluation of a multidisciplinary Training-of-Trainers programme designed to support school-based sexuality education within the ESPRIT project. Participants who completed the programme achieved higher knowledge scores than untrained participants at follow-up. However, findings should be interpreted cautiously given the exploratory design, small sample size, absence of baseline assessment, and use of a non-validated questionnaire. Further studies using larger samples, validated outcome measures, and longer follow-up periods are needed to evaluate programme effectiveness and implementation in real-world school settings.

## Figures and Tables

**Table 1:** Questionnaire on basic knowledge on sexuality education and health and responses in the experimental and comparator groups.

QUESTIONS	ANSWER OPTIONS	EXPERIMENTAL GROUP Correct Answer N° (%)	COMPARATOR GROUP Correct Answer N° (%)	*p*
What does the term FEMIDOM mean?	A sexual practice A sex toy **A contraceptive method** An emergency therapy	9 (90%)	5 (50%)	0.050
Which generation does a person born after 2012 belong to?	Y Z **Alpha** Beta	10 (100%)	0 (0%)	<0.0005
The setting in its components is analysed by a tool called	**GAS** SSC WPATH U = U	4 (40%)	3 (30%)	0.639
A trans man is a person whose sex assigned at birth was male	True **False**	9 (90%)	3 (30%)	0.006
In ICD-11, trans experiences are classified under which term?	Transsexualism Syndrome Gender identity disorder **Gender Incongruence** Gender Dysphoria	7 (70%)	1 (10%)	0.006
What does U = U mean?	It represents a typology of sexuality **It is a scientific slogan** It is a reference to femininity It is an evaluation tool	7 (70%)	4 (40%)	0.178
What is the most appropriate term to refer to a person living with HIV?	HIV-positive person **Person living with HIV** Positive person Person who has seroconverted	7 (70%)	4 (40%)	0.178
What is PEP?	A Diagnostic Protocol for STIs A Prevention Tool for Unprotected Sex **An Emergency Therapy** Polygamy Extended to Other Partners	6 (60%)	6 (60%)	1.0
Who developed the first theoretical model of the human sexual response cycle?	Bandura et al. **Master & Johnson** Kinsley & Hardy Goleman et al.	9 (90%)	1 (10%)	<0.0005
What is a dental dam?	An emergency contraceptive tool An oro-genital lesion **A barrier method for some STIs** A sex toy	9 (90%)	5 (50%)	0.050

In bold correct answer. Note: Items were selected to reflect the multidisciplinary contents addressed during the Training-of-Trainers programme, including sexual and reproductive health, STI prevention, HIV-related concepts, inclusive sexuality education, gender diversity, and communication with adolescents. Percentages refer to the proportion of participants providing the correct answer for each item.

## Data Availability

The datasets generated and/or analysed during the current study are available from the corresponding author on reasonable request.

## Supplementary Materials

The following supporting information can be downloaded at: https://www.mdpi.com/article/10.3390/ijerph23070843/s1, Table S1: Training activities and Learning Objectives—Day 1; Table S2: Training Activities and Learning Objectives—Day 2; Table S3: Training Activities and Learning Objectives—Day 3.
