# Supplementary material for: Development and Pilot Evaluation of a Training-of-Trainers Model for School-Based Sexuality Education Within the ESPRIT Project
Source: Int J Environ Res Public Health. 2026 Jun 26;23(7):843. doi: 10.3390/ijerph23070843 (PMC13410090; doi:10.3390/ijerph23070843)
Supplement: Supplementary file 1 [file ijerph-23-00843-s001.zip › ijerph-4343328-supplementary.pdf]

SUPPLEMENTARY MATERIAL

Table S 1- Training activities and Learning Objectives – Day 1

ACTIVITY	DESCRIPTION	LEARNING OBJECTIVE(S)
**Paired Introductions**	Participants were divided into pairs and given approximately 20 minutes to share personal experiences and relevant life events. At the end of the activity, each participant introduced their partner to the group by speaking in the first person, as if they were that individual.	Develop active listening, empathy, and interpersonal communication skills.
**Photo-Based Reflection Activity**	Participants were shown photographs portraying individuals with meaningful personal stories. Based solely on the images, participants were invited to share a word, emotion, or reflection. The facilitators then revealed the stories behind the photographs and guided a group discussion.	Challenge stereotypes and promote critical reflection on prejudice, assumptions, and first impressions.
**Working with Generation Alpha and Schools**	Theoretical background and practical applications related to Generation Alpha and school-based educational settings.	Increase understanding of the target population and the educational context in which sexuality education activities are delivered.
**Agree/Disagree Activity**	Participants stood along a line and responded to a series of statements by moving to one side if they agreed, or to the opposite side if they disagreed. Statements were related to topics addressed within the ESPRIT project.	Encourage reflection, promote discussion, and increase awareness of diverse perspectives and opinions.

Table S2. Training Activities and Learning Objectives – Day 2

ACTIVITY	DESCRIPTION	LEARNING OBJECTIVE(S)
Brainstorming	Structured group brainstorming focused on sexuality education topics and project-related activities.	Develop collaborative problem-solving and idea-generation skills.
Role-Playing Activities	Facilitators acted as teachers, while participants were randomly assigned different student roles (e.g., shy student, bully, class leader, student with a crush on the teacher). Participants engaged in simulated classroom situations related to sexuality education.	Develop empathy, communication skills, and the ability to manage diverse classroom dynamics.
Techniques for Leading Adolescent Groups	Presentation and discussion of group management techniques and peer education approaches, with particular attention to Generation Z and Generation Alpha.	Acquire practical skills for facilitating adolescent groups and implementing peer education strategies.
30 Seconds in the Spotlight	Participants sat in a circle while a facilitator presented a series of sexuality-related terms. One participant at a time was invited to sit in the centre and continue a collective story using the assigned term. Every 30 seconds, a new participant took over and contributed to the evolving narrative. At the end of the activity, all terms and concepts were discussed and clarified.	Reinforce sexuality-related terminology and concepts, expand participants' vocabulary, and promote confidence in public speaking through an interactive storytelling exercise.
Genderbread Person Activity	Using the "Genderbread Person" model, facilitators introduced and discussed key dimensions of sexual identity, including gender identity, gender expression, biological sex, and sexual and romantic attraction.	Enhance understanding of gender identity, gender expression, biological sex, and sexual and romantic attraction while promoting inclusivity and respect for diversity.
Icebreaker Activity	Participants were each given a glass of water. Unbeknownst to the group, two facilitators added salt to their own glasses before the activity began. While music played, participants exchanged small amounts of water with one another during repeated "toasts." After several rounds, participants tasted the water remaining in their own glass and observed differences in salinity. The activity was subsequently used to illustrate the transmission dynamics of infectious diseases.	Demonstrate how infectious diseases can spread through social interactions using a simple experiential learning activity that can be replicated in school settings.
Step Across the Line	Participants positioned themselves individually within the room and were invited to imagine a line in front of them. The facilitator read a series of statements, and participants could choose to step across the line if they identified with the statement or remain in place if they did not wish to respond. Participation was entirely voluntary.	Promote self-reflection, emotional awareness, and exploration of personal experiences within a safe training environment.
Introduction to Social Design	Introduction to social design principles, project planning, activity scheduling, time management, and subgroup practical exercises.	Develop teamwork, project planning, and organizational skills for designing educational interventions.

Table S3. Training Activities and Learning Objectives – Day 3

ACTIVITY	DESCRIPTION	LEARNING OBJECTIVE(S)
Sharing Completed Projects	Each subgroup presented the project they had developed to the group through a PowerPoint presentation.	Apply the knowledge and skills acquired during the training programme through collaborative project presentations.
Project Clarification and Open Questions	Facilitators provided guidance and clarification regarding the implementation of school-based activities, including intervention procedures, timelines, and organizational aspects.	Clarify implementation procedures, timelines, and practical aspects of school-based activities.
"I Thank the Group Because…" and Closing Reflections	Participants were invited, on a voluntary basis, to stand at the centre of the group and complete the sentence "I thank the group because…", sharing reflections on their training experience. The activity concluded the training programme and provided an opportunity for collective reflection.	Encourage reflection, group cohesion, and closure of the training experience.
